# Correction: Steele et al. Misincorporation Proteomics Technologies: A Review. *Proteomes* 2021, *9*, 2

**DOI:** 10.3390/proteomes10020022

**Published:** 2022-06-16

**Authors:** Joel R. Steele, Carly J. Italiano, Connor R. Phillips, Jake P. Violi, Lisa Pu, Kenneth J. Rodgers, Matthew P. Padula

**Affiliations:** 1 Proteomics Core Facility and School of Life Sciences, The University of Technology Sydney, Ultimo, NSW 2007, Australia; joel.steele@uts.edu.au (J.R.S.); jake.violi@uts.edu.au (J.P.V.); 2 Neurotoxin Research Group, School of Life Sciences, The University of Technology Sydney, Ultimo, NSW 2007, Australia; carly.italiano@uts.edu.au (C.J.I.); connor.phillips@student.uts.edu.au (C.R.P.); lisa.pu@student.uts.edu.au (L.P.); kenneth.rodgers@uts.edu.au (K.J.R.)

## Error in Table

In the original publication, there was a mistake in Table 2 as published [1]. There were spelling/typographical errors that led to incorrect information to align to the wrong amino acid. Therefore, unfortunately the assignments are incorrect. We have restored the correct assignments and ensured the fidelity of the table. The core idea behind the table is unchanged by the correction, as it was only used to provide examples of PTMs that are found on amino acids; as such, the correct reformatting does not impact the concepts/ideas/message provided by this review in any way. The corrected Table 2 appears below.

The authors apologize for any inconvenience caused and state that the scientific conclusions are unaffected. The original publication has also been updated.

## Figures and Tables

**Table 2:** Amino acids and their biological post-translational modifications.

Site of Modification	Letter Symbol	Modification
Alanine	A	Carbonylation
Arginine	R	Hydroxylation, Phosphorylation, Methylation, ADP-ribosylation, Citrullination, Carbonylation
Asparagine	N	Hydroxylation, Methylation, N-linked glycosylation
Aspartic acid	D	Hydroxylation, Phosphorylation, Methylation
Cysteine	C	Hydroxylation, Phosphorylation, Methylation, Sulfation, Myristoylation, ADP-ribosylation, Nitrosylation
Glutamic acid	E	Phosphorylation, Methylation, ADP-ribosylation
Glutamine	Q	Methylation, Carbonylation
Glycine	G	Myristoylation
Histidine	H	Phosphorylation, Methylation
Isoleucine	I	Methylation, Carbonylation
Leucine	L	Methylation
Lysine	K	Hydroxylation, Phosphorylation, Methylation, Ubiquitination, Myristoylation, ADP-ribosylation, Carbonylation, Malonylation, Succinylation, Glutarylation, Biotinylation
Methionine	M	Hydroxylation
Phenylalanine	F	Hydroxylation
Proline	P	Hydroxylation
Serine	S	Phosphorylation, Methylation, Sulfation, O-linked glycosylation, Carbonylation, Decanoylation
Selenocysteine	U	Hydroxylation
Threonine	T	Phosphorylation, Methylation, Sulfation, O-linked glycosylation, Decanoylation
Tryptophan	W	Glycosylation, Bromination, Quinone
Tyrosine	Y	Hydroxylation, Phosphorylation, Sulfation, O-linked glycosylation, Quinone
Valine	V	Hydroxylation, Carbonylation

The table provides context for how a misincorporation at a particular site could have large biological impacts on cell function [58].
